# Electrodeposition of Hybrid Magnetostrictive/Magnetoelectric Layered Systems

**DOI:** 10.3390/ma14216304

**Published:** 2021-10-22

**Authors:** Sara Abad, Alicia Prados, Marco Maicas, Neven Biskup, Maria Varela, Rocio Ranchal

**Affiliations:** 1 Departamento Física de Materiales, Facultad Ciencias Físicas, Universidad Complutense de Madrid, Plaza de las Ciencias 1, 28040 Madrid, Spain; sarabad@ucm.es (S.A.); aliciapradosdiaz@ucm.es (A.P.); nbiskup@pdi.ucm.es (N.B.); mvarela@fis.ucm.es (M.V.); 2 Institute for Optoelectronic Systems and Microtechnology, Polytechnic University of Madrid, Avenida Complutense 30, 28040 Madrid, Spain; marco.maicas@upm.es; 3 Instituto Pluridisciplinar, Universidad Complutense de Madrid, Paseo Juan XXIII 1, 28040 Madrid, Spain; 4 Instituto de Magnetismo Aplicado, Universidad Complutense de Madrid-Adif-Consejo Superior de Investigaciones Científicas, P.O. Box 155, 28230 Las Rozas, Spain

**Keywords:** electrodeposition, phase segregation, hybrid layered

## Abstract

The potential use of electrodeposition to synthesize a hybrid magnetostrictive/magnetoelectric layered system is shown in this paper. By appropriately adjusting pH, growth potential, and electrolyte composition, it is possible to achieve thin films in which magnetoelectric oxide GaFeO_3_ (GFO) is formed in close contact with magnetostrictive metallic FeGa alloy. X-ray diffractometry shows the formation of FeGa as well as GFO and Fe oxides. Electron microscopy observations reveal that GFO mainly segregates in grain boundaries. Samples are ferromagnetic with an isotropic magnetic behavior in the sample plane. Magnetic stripes are observed by magnetic force microscopy and are correlated to Fe_3_O_4_. When its segregation is minimal, the absence of stripes can be used to monitor Fe oxide segregation.

## 1. Introduction

Magnetoelectric materials have received a great deal of interest in the last few years partly because of their potential applications in nanotechnology [1]. One of the most attractive possibilities of magnetoelectric materials is magnetoelectric random access memories (MeRAM) [2], in which information is stored through electric polarization and recording is magnetically performed. In this way, a decrease in energetic consumption is expected in comparison to actual memories [3]. 

The magnetoelectric properties of GaFeO_3_ (GFO) oxides were discovered in the 1960s in bulk samples [4,5]. Due to the interest in multiferroic thin films, GFO oxides gained renewed interest in the 2000s [6,7,8,9,10]. GFO oxides have an orthorhombic Pc2_1_n structure (group number 33) for the composition range in which magnetoelectricity is observed [5]. The magnetic order is ferrimagnetic, and the magnetoelectric constant, α = ∂P/∂H, where P is the electric polarization and H the applied magnetic field, can reach a value of around 20 ps/m. Due to this large α, the development of GFO-based MeRAMs is one of its most promising applications and can represent a breakthrough for this material system that is eco-friendly because of its free, rare-earth composition.

GFO has been synthesized using different techniques, such as the floating-zone method [6], sol–gel [11,12,13], solid state route [14,15,16,17,18] and solid-state chemical reaction [19]. High-quality, thin films have been obtained when using pulsed laser deposition (PLD) [7,20,21,22,23], but industrial methods are needed for massive production. The inherent problems for the synthesis of this material system were highlighted when shown the necessity of a large optimization process in the solid-state route [14], and problems were detected when trying to electrodeposit GFO [24]. Electrodeposition is a technique routinely used by the industry, but there is a lack of papers devoted to this issue for GFO. Very recently, the inhibition of Fe oxidation has been shown when trying to electrodeposit GFO; indications of oxidation were only detected for Ga, whereas Fe remained in its metallic state [24]. This is in agreement with previous results obtained when studying the effect of thermal treatments in oxygen atmosphere on FeGa thin films [25]. In that work, while Ga was strongly oxidized and eventually evaporated as the oxidation temperature was raised, Fe remained in its metallic state.

In a paper of K. S. M. Reddy et al., the appearance of GFO oxides was reported when trying to electrodeposit FeGa alloys from citrate-based electrolytes for rotation rates (√ω) lower than 15 min^1/2^ [26], and the presence of oxygen was also detected in another independent work for non-optimized growth conditions using a magnetically stirred electrolyte during the electrodeposition of metallic FeGa alloys [27]. Therefore, the formation of GFO seems to be possible during the electrodeposition of FeGa.

One of the issues to be solved in MeRAMS is how to maintain the induced magnetic anisotropy when the electric field is removed [28]. Therefore, in this work, we explored the possibility of using electrodeposition to synthetize a hybrid magnetostrictive/magnetoelectric layer comprised of FeGa (magnetostrictive) and GFO (magnetoelectric). In this way, the magnetostrictive material can maintain the remanent magnetization state induced by the electric field applied in the magnetoelectric layer. We took as a reference our previous study about GFO electrodeposition [24] to explore growth conditions for the synthesis of a hybrid layer. We successfully observed metallic FeGa with GFO segregated at the grain boundaries. Thus, this work shows the potential use of electrodeposition to synthesize systems with potential application in the development of MeRAMS.

## 2. Materials and Methods

Samples were electrodeposited on top of Si substrates that were metallized to increase their electric conductivity with 10 nm of Ti followed by 100 nm of Au. FeSO_4_ and Ga_2_(SO_4_)_3_ were the source of Fe^2+^ and Ga^3+^ ions, respectively; Na_3_-citrate was used as an antioxidant; and Na_2_SO_4_ was used to increase the electrolyte conductivity. Concentration of citrate (50 mM), Na_2_SO_4_ (500 mM), and Ga_2_(SO_4_)_3_ (35 mM) was the same in all the electrolytes, whereas FeSO_4_ ranged between 12 and 35 mM. Table 1 summarizes the growth conditions in terms of electrolyte composition, growth potential, and pH. Electrolytes were prepared with deionized water, and pH was adjusted by means of diluted NH_4_OH (10% vol.). A PalmSens EmStat3+Blue potentiostat was used to control the electrodeposition processes that were carried out in a three-electrode cell with a platinum mesh as a counter electrode and an Ag/AgCl (3 M NaCl) reference electrode from Bioanalytical Systems, Inc. (West Lafayette, IN, USA) (*E_eq_* = 0.196 V vs. SHE). Electrochemical experiments were performed without agitation or magnetic stirring in nonrotating substrates at 300 K. The nominal thickness of the films (200 nm) was adjusted by means of Faraday’s law [24].

**Figure 1:**
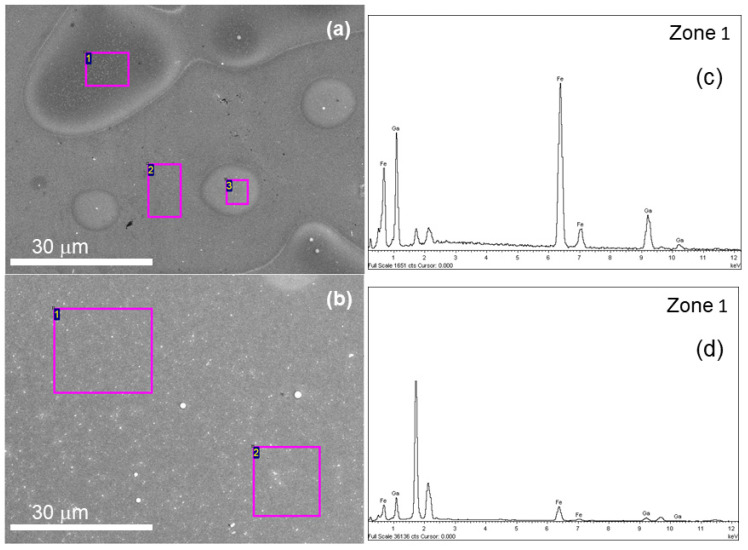
SEM images for samples grown with electrolytes III and IV for a potential of −1.150 V. (**a**) Electrolyte III. Composition measured by EDS in each zone is: zone (1) 64 at.% Fe, 36 at.% Ga; zone (2) 90 at.% Si, 10 at.% Au; zone (3): 62 at.% Fe, 38 at.% Ga. (**b**) Electrolyte IV. Composition measured by EDS in each zone is: zone (1) 59 at.% Fe, 41 at.% Ga; zone (2) 58 at.% Fe, 42 at.% Ga. (**c**,**d**) are EDS spectrum of zone 1 of (**a**,**b**) images, respectively.

X-ray diffractometry (XRD) in the Bragg–Brentano configuration was performed in a Philips X’Pert MPD using the Cu K_α_ wavelength (1.54056 Å). Diffraction patterns were measured with an angle step of 0.026°. The surface morphology was imaged with a JEOL JSM 6400 scanning electron microscope (SEM) operated at 20 kV and 80 mA. The same working conditions were used in the SEM microscope to measure the Fe and Ga content of the samples by means of energy dispersive X-ray spectroscopy (EDS). Typically, we measured from 3 to 4 different regions in the same sample to confirm its compositional homogeneity. The measured area can be slightly different from zone to zone, but it ranged from 20 μm^2^ to 10 μm^2^ as seen in Figure 1. Scanning transmission electron microscopy (STEM) images were taken in a JEOL ARM200 cF microscope operated at 200 kV. The nanometer-sized chemical characterization was carried out by electron energy-loss spectroscopy (EELS) using a Gatan “Quantum” spectrometer. The analysis of the EELS data was performed using the routines available through the Gatan “Digital Micrograph” software.

At room temperature, in-plane and perpendicular hysteresis loops were measured in a vibrating sample magnetometer (VSM). The data were normalized to the highest magnetization value of the corresponding loop. The size of the experimental points is larger than the precision of the measurement. An atomic force microscope (AFM) working in tapping mode was used to measure the roughness of the layers using the root mean square (rms) to quantify it. Magnetic force microscopy (MFM) was used to analyze the magnetic contrast measured using the phase detection mode, i.e., monitoring the cantilever’s phase of oscillation, while the magnetic tip was scanning the sample surface at a distance of 40 nm on average (lift mode). We used a multimode AFM from Digital Instruments. The cantilevers were from Veeco and the tips were coated with Co/Cr with a resonant frequency of 75 kHz and a nominal force constant of 2.8 N/m. We used the double scan method: in the first scan, we measured the topography, and in the second scan, we lifted the tip to obtain the magnetic contrast as commented above. The MFM measurements were performed at remanence after an in-plane magnetic field of 8 kOe was applied. AFM and MFM images were analyzed with WSxM 5.0 software and Nanoscope 5.31r1 software.

## 3. Results and Discussion

The objective of this work is to explore the capability of the electrodeposition technique to synthesize an FeGa/GFO layered system. First, we focused our attention on the EDS measurements to determine the Fe/Ga ratio in the samples (Table 1). Starting with a comparison between electrolytes with a fixed pH of 4, it can be observed in Table 1 that the composition of the samples can be tuned by means of growth potential. For each electrolyte, there is a decrease in the amount of Fe in the layers when the potential decreases from −1.075 V to −1.150 V. The modification of the FeSO_4_ content from 35 mM to 12 mM has a negligible effect on the Fe/Ga ratio in the deposit for −1.075 V, whereas it is reduced from 69 at.% to 64 at.% for a potential of −1.150 V, and from 76 at.% to 70 at.% for −1.100 V. Increasing the pH to 8 (electrolyte IV) further reduces the Fe content in the electrodeposited layers, reaching a minimum Fe/Ga ratio of 58/42. For a pH of 8 and a potential of −1.075 V, no layer was deposited. Therefore, the total amount of Fe and Ga inside the deposited films can be tuned by means of the potential, FeSO_4_ in the electrolyte, and pH.

**Figure 2:**
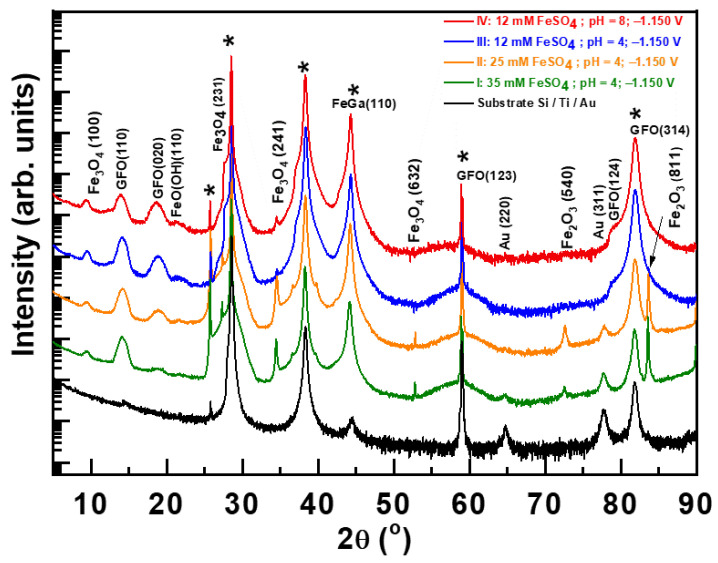
X-ray diffractometry patterns of samples deposited with all of the studied electrolytes at −1.150 V. Curves are vertically shifted for clarity. Diffraction peaks labelled with * are related to the Si/Ti/Au substrate.

In Figure 2, we compare the diffraction patterns for all the samples deposited at −1.150 V. For the identification of the diffraction peaks, we used the following XRD cards: Fe_3_O_4_ (01-076-0958), GFO (01-082-9300), FeO(OH) (04-014-5919), and Fe_2_O_3_ (00-039-1346 and 00-039-0238). FeGa has the same BCC structure as pure Fe but with sifted peaks due to the introduction of Ga in the Fe lattice [24,27]. As evidenced by XRD, apart from metallic FeGa, the experiment formed GFO, Fe oxides (Fe_3_O_4_: magnetite and γ-Fe_2_O_3_: maghemite), and Fe hydroxide (FeO(OH): goethite) (Figure 2). To better understand the evolution of the segregation, we integrated the area below each diffraction peak by means of Origin software (Table 2). In the integrated areas related to GFO (110), (020), and (124), reflections increase as the content of FeSO_4_, i.e., Fe^2+^ in the electrolyte, decreases (Table 1 and Table 2). At the same time, there is a decrease in the integrated areas of Fe_3_O_4_ (100), (231), (241), and (632), and of Fe_2_O_3_ (540) and (811). Therefore, the content of the Fe^2+^ in the electrolyte tunes Fe oxide segregation. The smaller the Fe/Ga ratio in the layers, the higher the formation of the GFO and the lower formation of Fe oxides. Fe_2_O_3_ is a product of the GFO decomposition in oxygen deficiency conditions, and, in fact, there is a region of coexistence of Fe_2_O_3_-Fe_3_O_4_ in the ternary system and Fe_2_O_3_-FeO-Ga_2_O_3,_ from which GaFeO_3_ can be formed [29]. Then, the conditions favorable for GFO formation reduce the segregation of Fe oxides. In fact, when comparing electrolytes I to III, it is significant that the appearance of the GFO (124) reflection for electrolyte III reflects an enhancement of GFO formation, whereas at the same time, some Fe oxide reflections are absent: Fe_3_O_4_ (632), Fe_2_O_3_ (540), and Fe_2_O_3_ (811). FeO(OH) hydroxide is increased with the same conditions as GFO (Table 2), a result that can be understood considering that it is a precursor of FeO [30]. As GFO can be formed from FeO, the hydroxide acts as an intermediate for GFO segregation.

Comparing samples from electrolytes III and IV provides insight into the effect of pH (Table 2). In this sense, electrolyte IV increases the integrated area of GFO (020) and (124) diffraction peaks, whereas it decreases that of GFO (110). This points in the direction of better crystallinity for electrolyte IV. In the morphology of the samples obtained with these two electrolytes, when studied using the SEM, there are notable differences that can be observed. Whereas the substrate from electrolyte III is not completely covered, even after a deposition time enough for a 200 nm nominal thickness, the sample from electrolyte IV exhibits complete coverage (Figure 1). With an AFM, it was possible to measure a root mean square (rms) roughness of 13 nm for electrolyte IV, whereas it was not possible to infer it for samples deposited with electrolyte III due to the large degree of inhomogeneities. The rms, as well as SEM images for electrolytes I and II, is summarized in the Supplementary Information. 

**Figure 3:**
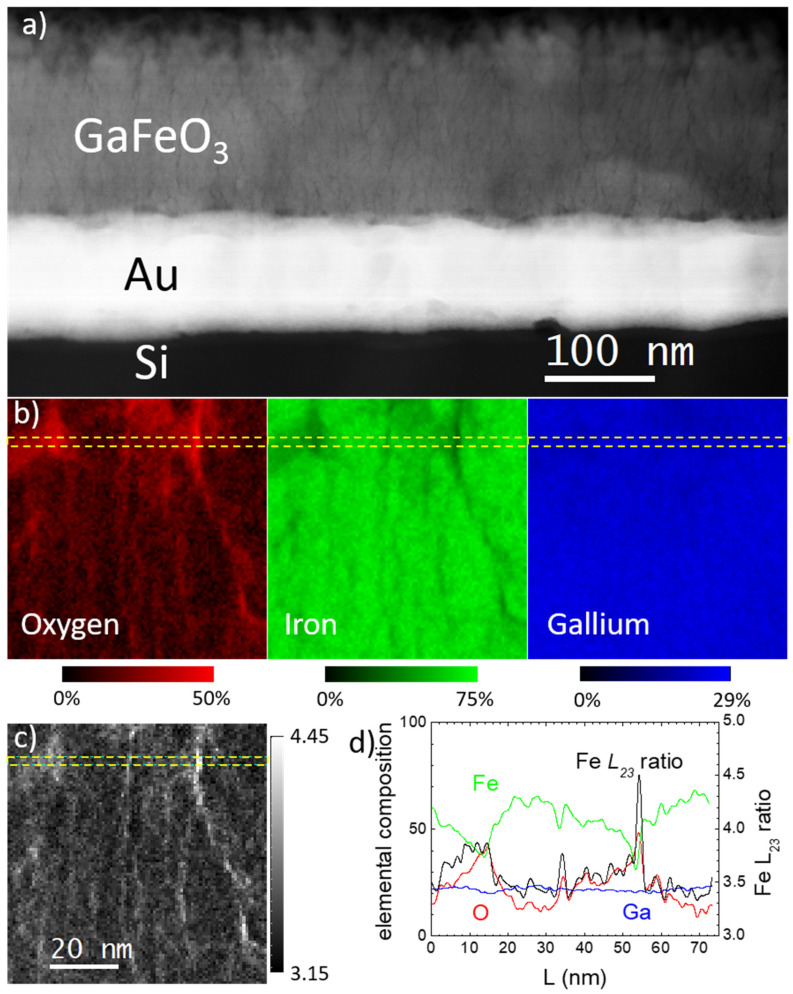
(**a**) Scanning transmission electron microscopy high-angle annular dark-field image of the FeGa/GaFeO_3_ thin film. (**b**) Elemental maps showing the local Ga, Fe, and O at.% compositions of a 74 nm × 74 nm area of this film. (**c**) Iron *L*_23_ ratio measured in this area. (**d**) The profiles of local compositions along with *L*_23_ ratios along the directions marked with yellow dashed lines on the adjacent maps.

We examined the samples by STEM to gain a deeper insight into the segregation process itself. Figure 3 shows a cross-section view of the film of FeGa/GaFeO_3_ grown on the silicon substrate coated with Au and Ti. Figure 3a displays a high-angle annular dark-field image (HAADF) of the film. For this imaging mode, also known as Z-contrast, the local contrast is roughly proportional to Z^2^ (Z being the atomic number), so heavier atoms/elements appear brighter and light atoms appear dimmer. The film thickness is 200 nm, as expected. The Au buffer is clearly observed, while the film itself exhibits an inhomogeneous contrast with a dense network of filaments or regions with a darker contrast, which points to an uneven composition. This apparent chemical inhomogeneity was checked using EELS. Combining STEM and EELS, we can correlate the segregation of GFO to the dark areas observed in the TEM images. Figure 3b shows relative atomic chemical compositional maps based on the analysis of O *K*, Fe *L*_2,3_ and Ga *L*_2,3_ EELS edges. According to these data, the regions appearing as dark channels or filaments are clearly O rich and, therefore, likely to be comprised of GaFeO_3_, while the rest of the sample seems closer to a Ga/Fe alloy. A profile of the compositional maps along the direction indicated with dashed yellow lines in Figure 3b is shown in Figure 3d. From the compositional profile, it can be inferred that Fe is the dominant element in the areas of the sample that appear brighter on the HAADF image (with composition values up to atomic 70% in some regions), while the Ga content is approximately even with values around 22%. Only in the areas that look like dark channels does the oxygen signal increase significantly—up to 50%, in fact. Knowing that this apparent composition is averaged down the whole sample thickness (approximately 20–30 nm), and that the channel morphology may change at different depths within the sample, we can conclude that these dark regions are indeed consistent with the local presence of GaFeO_3_, where the atomic O composition should reach 60%. This finding is in accordance with the iron oxidation state map shown in Figure 3c. It is known that the *L*_23_ intensity ratio—the ratio between the intensity of the *L*_3_ and *L*_2_ edges—of transition metals depends on the oxidation state of these atoms [31,32]. Iron has the highest *L*_23_ ratio when it contains five electrons in the 3*d* shells. This occurs in the presence of Fe^3+^, the expected oxidation state of iron in GaFeO_3_. The increase in the Fe *L*_23_ ratio in the areas that look like dark channels corroborates the assignment of these filaments as GaFeO_3_ rich. Such inhomogeneous GFO segregation can perhaps be understood considering that they are related to grain boundaries where the electric current density is much higher, making GFO electrodeposition easier in these regions due to its low electric conductivity.

**Figure 4:**
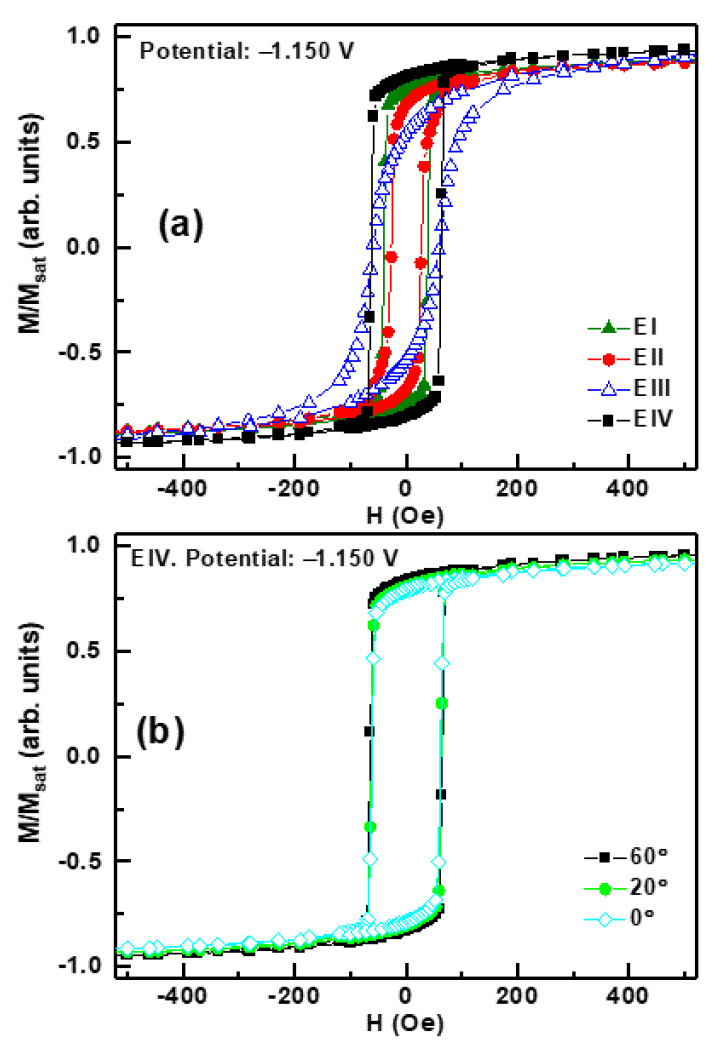
(**a**) In-plane hysteresis loops at room temperature for samples deposited at −1.150 V: (▲) electrolyte I, (●) electrolyte II, (∆) electrolyte III, and (■) electrolyte IV. (**b**) In-plane hysteresis loops at room temperature for the sample deposited at −1.150 V with electrolyte IV. The magnetic field was applied in the sample plane at different angles between a reference direction, which is taken as 0°: (■) 60°, (●) 20°, and (◊) 0°.

The in-plane hysteresis loops reflect a ferromagnetic behavior with no magnetic anisotropy in the studied samples (Figure 4). The coercive field (*H_C_*) and the *M_r_*/*M_max_* ratio between the magnetic remanence (*M_r_*) and the magnetization at the maximum applied magnetic field (*M_max_*), also defined as squareness, can be used to quantitatively monitor the evolution of the in-plane magnetic properties. To calculate the *H_C_,* we performed linear interpolations of the experimental data. In Table 3, we summarize these two magnetic parameters (*M_r_*/*M_max_* and *H_C_*) for in-plane measurements, together with the integrated area below FeGa and GFO diffraction peaks, growth potential, and electrolytes to better compare the results. It is also important to recall the magnetic behavior for each of the phases present in the samples: FeGa, Fe_2_O_3_, and Fe_3_O_4_ are ferromagnetic [33,34]; GFO is ferrimagnetic [6,7]; and FeO(OH) is antiferromagnetic [35]. Therefore, the existence of remanence and coercivity in the hysteresis loops can be due to all of them except for FeO(OH). 

Comparing samples for each electrolyte, the in-plane *M_r_*/*M_max_* ratio increases with the integrated area below the FeGa (110) diffraction peak. The same trend is observed when analyzing samples deposited at −1.150 V for different electrolytes. Therefore, in-plane magnetic behavior seems to be governed by FeGa. On the other hand, coercivity increases with the proportion of GFO (Table 3), and this can be correlated to the coupling between FeGa and GFO. 

**Figure 5:**
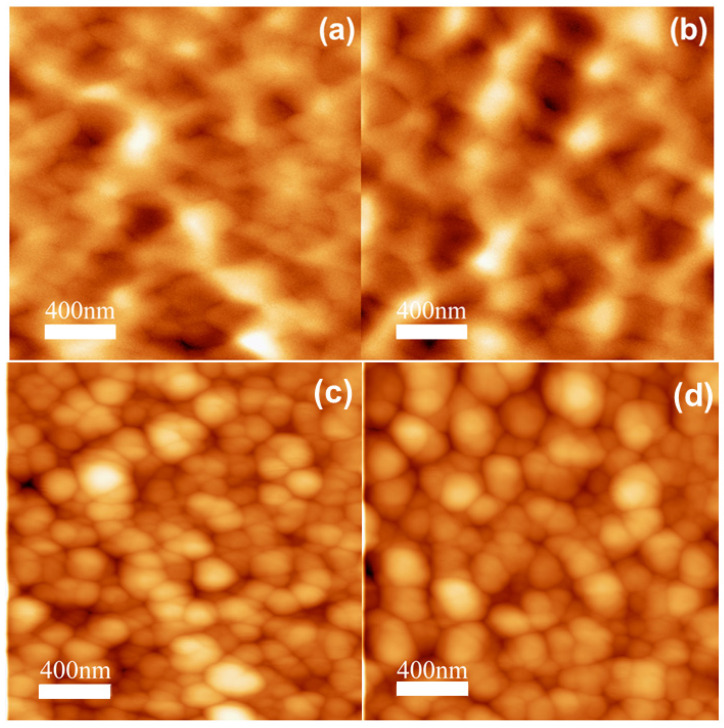
(2 × 2) μm^2^ MFM images recorded at remanence for samples deposited at −1.150 V: (**a**) electrolyte I, (**b**) electrolyte II. (**c**,**d**) (2 × 2) μm^2^ AFM images of samples of (**a**,**b**), respectively.

**Figure 6:**
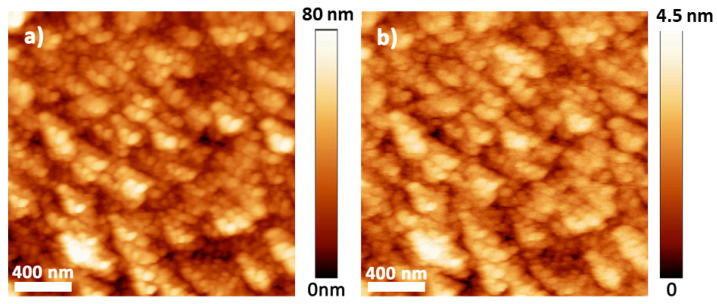
(2 × 2) μm^2^ images of the same area recorded with (**a**) AFM and (**b**) MFM for the sample deposited at −1.150 V with electrolyte IV.

We also characterized the out-of-plane magnetic properties combining magnetometry and MFM. Notably, we clearly recorded a magnetic contrast that reveals the presence of magnetic stripes in samples deposited with electrolytes I and II (Figure 5a,b). The comparison between MFM and AFM images reflects that magnetic contrast is not related to topographical features (Figure 5). Samples with electrolyte III could not be imaged because of their large roughness, and samples from electrolyte IV did not exhibit stripes (Figure 6). It is well known that a thin ferromagnetic film displaying moderate perpendicular magnetic anisotropy (PMA) can experience the competition between the PMA energy, which promotes local alignment along the out-of-plane direction, and long-range magnetostatic energy, which favors in-plane magnetization, resulting in magnetic stripe domains [36,37,38].

To track the evolution of the out-of-plane magnetic behavior, we recorded the out-of-plane hysteresis loop and inferred the *M_r,perp_/M_max,perp_* ratio from them (Table 3). In general, the *M_r,perp_/M_max,perp_* ratio is quite small, even for samples deposited from electrolytes I and II, and for samples that exhibit magnetic stripes. We can understand the small *M_r,perp_/M_max,perp_* ratio because in the hysteresis loops, there is a magnetic contribution from all of the magnetic phases present in the layers. Thus, the perpendicular squareness is small, because the magnetization is almost in the sample plane.

Stripes observed by MFM are a result of the presence of phases with a moderate PMA. Stripes have been previously reported in Fe_3_O_4_ [39,40], whereas FeGa generally presents a magnetic contrast known as ripple [33,41,42], and to the best of our knowledge, there are no published works about stripes in Fe_2_O_3_ or GFO. Since magnetic stripes are present in the samples with the highest proportion of Fe_3_O_4_, they can be used to track Fe_3_O_4_ segregation (Table 2). In fact, since Fe_3_O_4_ and Fe_2_O_3_ are minimized in the same growth conditions, magnetic stripes appear as a signature of Fe oxide segregation. Therefore, we found that growth conditions for electrolyte IV (FeSO_4_: 12 mM, Ga_2_(SO_4_)_3_: 35 mM, citrate: 50 mM, Na_2_SO_4_: 500 mM, pH 8) are optimized to reduce Fe oxide segregation and enhance GFO formation. Taking into account all of the results presented in this work, electrodeposition appears to be a very promising technique to synthesize hybrid magnetostrictive/magnetoelectric layered systems.

## 4. Conclusions

We explored the use of the electrodeposition technique to tune phase segregation as a way to synthetize hybrid magnetostrictive/magnetoelectric layered systems. The difficulties in Fe oxidization when using electrolytes to grow GFO were exploited to fabricate metallic magnetostrictive FeGa, with magnetoelectric GFO segregated in the shape of filaments. Phase segregation can be tuned by means of growth conditions in terms of electrolyte composition, growth potential, and pH. Samples exhibited ferromagnetism with isotropic behavior in the sample plane that was correlated with FeGa. Magnetic stripes were observed in samples with a higher Fe_3_O_4_ proportion. STEM–EELS observations revealed that GFO mainly segregates in the grain boundaries, possibly serving as a point of close contact between magnetostrictive and magnetoelectric phases. Minimal Fe oxide segregation was found for optimized electrodeposition conditions.

## Figures and Tables

**Table 1:** Summary of the growth conditions in terms of composition of FeSO_4_ in the electrolyte, pH, and growth potential. The Fe/Ga ratios measured in the samples by EDS are also added.

Elec.	FeSO_4_ (mM)	pH	Potential (V)	Fe/Ga Ratio (at. %)
I	35	4	−1.150	69/31
			−1.100	76/24
			−1.075	77/23
II	25	4	−1.150	66/34
			−1.100	70/30
			−1.075	77/23
III	12	4	−1.150	64/36
			−1.100	70/30
			−1.075	76/24
IV	12	8	−1.150	58/42
			−1.100	66/34
			−1.075	-

**Table 2:** Integrated areas below diffraction peaks for samples deposited at −1.150 V for all the studied electrolytes.

2*θ*	Compound	hkl	Integrated Area			
			I	II	III	IV
9.33	Fe_3_O_4_	011	635	476	396	365
14.04	GFO	110	1264	1697	1793	1257
18.59	GFO	020	147	974	1018	1160
21.21	FeO(OH)	110	0	44	92	117
27.36	Fe_3_O_4_	231	1530	1667	11	12
34.14	Fe_3_O_4_	241	2795	2968	24	53
44.30	FeGa	110	16,206	74,219	42,129	57,530
52.85	Fe_3_O_4_	632	25	21	-	-
72.40	Fe_2_O_3_	540	52	175	-	-
78.86	GFO	124	-	-	152	163
83.63	Fe_2_O_3_	811	1939	1548	-	-

**Table 3:** Summary of the *H_c_* and squareness obtained from in-plane and perpendicular hysteresis loops together with the potential and electrolyte used for the samples’ growth. Integrated area below FeGa(110) and different GFO diffraction peaks are also presented.

		In Plane	In Plane	Perpendicular	Integrated Area Below Diffraction Peaks			
Elec.	Pot. (V)	*H_c_* (Oe)	*M_r_/M_max_* (arb. units)	*M_r,perp_/M_max,perp_* (arb. units)	FeGa (110)	GFO (110)	GFO (020)	GFO (124)
I	−1.150	26	0.67	0.03	16,206	1264	147	-
	−1.100	26	0.70	0.08	12,420	1097	974	-
	−1.075	19	0.65	0.13	16,795	995	566	-
II	−1.150	41	0.75	0.11	74,219	1697	975	-
	−1.100	30	0.60	0.08	36,783	1363	1382	-
	−1.075	34	0.12	0.02	1624	1700	970	-
III	−1.150	61	0.53	0.11	42,129	1793	1018	152
	−1.100	27	0.06	0.01	3136	1046	1304	98
	−1.075	49	0.05	0.01	578	946	897	79
IV	−1.150	61	0.78	0.03	57,530	1257	1160	163
	−1.100	60	0.80	0.14	55,426	1251	802	108
	−1.075	163	0.25	0.08	132	1243	1203	87

## Data Availability

The data presented in this study are available on request from the corresponding author.

## Supplementary Materials

The following are available online at https://www.mdpi.com/article/10.3390/ma14216304/s1, Figure S1: SEM images for samples grown with electrolytes I and II for a potential of −1.150 V. (a) Electrolyte I. (b) Electrolyte II. (c) and (d) are EDS spectrum of samples of (a) and (b), respectively.
