# Supplementary material for: Electrodeposition of Hybrid Magnetostrictive/Magnetoelectric Layered Systems
Source: Materials (Basel). 2021 Oct 22;14(21):6304. doi: 10.3390/ma14216304 (PMC8585353; doi:10.3390/ma14216304)
Supplement: Supplementary file 1 [file materials-14-06304-s001.zip › materials-1411833-supplementary.pdf]

Supplementary information

Electrodeposition of Hybrid Magnetostrictive/Magnetoelectric Layered Systems

Sara Abad [1], Alicia Prados [1], Marco Maicas [2], Neven Biskup [1,3], Maria Varela [1,3] and Rocio Ranchal [1,4,*]

[1] Departamento Física de Materiales, Facultad Ciencias Físicas. Universidad Complutense de Madrid, Plaza de las Ciencias 1, 28040 Madrid, Spain; sarabad@ucm.es (S.A.); aliciapradosdiaz@ucm.es (A.P.); nbiskup@pdi.ucm.es (N.B.); mvarela@fis.ucm.es (M.V.)
[2] Institute for Optoelectronic Systems and Microtechnology, Polytechnic University of Madrid, Avenida Complutense 30, 28040 Madrid, Spain; marco.maicas@upm.es
[3] Instituto Pluridisciplinar, Universidad Complutense de Madrid, Paseo Juan XXIII 1, 28040 Madrid, Spain
[4] Instituto de Magnetismo Aplicado, Universidad Complutense de Madrid-Adif-Consejo Superior de Investigaciones Científicas, P.O. Box 155, Las Rozas, Madrid 28230, Spain
* Correspondence: rociran@ucm.es

SEM images of samples deposited at -1.150 V for electrolytes I and II, respectively.

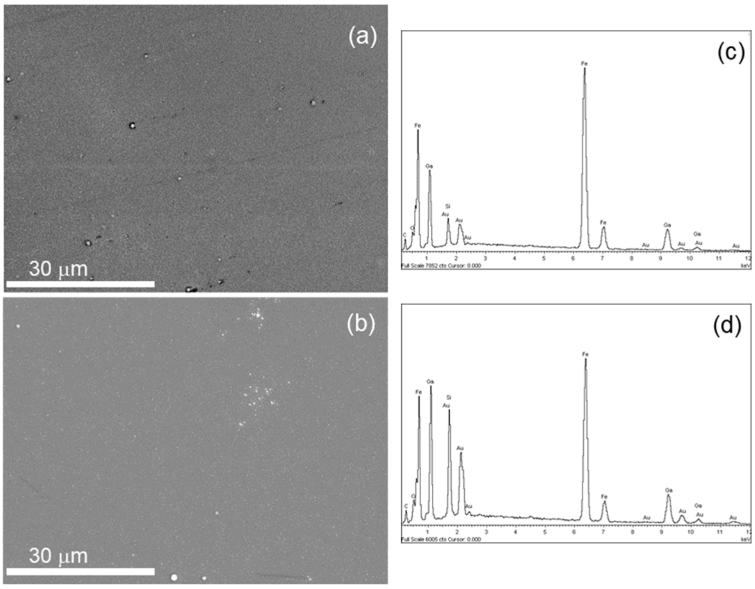

Figure S1. SEM images for samples grown with electrolytes I and II for a potential of -1.150 V. **(a)** Electrolyte I. **(b)** Electrolyte II. **(c)** and **(d)** are EDS spectrum of samples of (a) and (b), respectively.

Root-mean-square (rms) obtained from the AFM images for the samples studied in this work. rms for uncoated substrates is 3.4 nm.

Elec.	Potential (V)	rms (nm)
	-1.150	9.8
I	-1.100	14.0
	-1.075	11.3
	-1.150	9.7
II	-1.100	8.6
	-1.075	8.7
	-1.150	3D
III	-1.100	3D
	-1.075	3D
IV	-1.150	13.3

	-1.100	12.8
	-1.075	11.9
